# Impact of thermal treatment on halloysite nanotubes: A combined experimental-computational approach

**DOI:** 10.1016/j.heliyon.2024.e39952

**Published:** 2024-10-30

**Authors:** Ahmed Abotaleb, Ivan Gladich, Kamal Mroue, Nada Abounahia, Alaa Alkhateeb, Abdulaziz Al-Shammari, Yongfeng Tong, Dema Al-Masri, Alessandro Sinopoli

**Affiliations:** a Qatar Environment and Energy Research Institute, Hamad Bin Khalifa University, P.O. Box 34110, Doha, Qatar; b HBKU Core Laboratories, Hamad Bin Khalifa University, P.O. Box 34110, Doha, Qatar; c Earthna Center for a Sustainable Future, Qatar Foundation, Doha, Qatar

**Keywords:** halloysite, ^29^Si NMR, ^27^Al NMR, molecular dynamics, Calcination

## Abstract

Halloysite nanotubes (HNTs) are naturally occurring aluminosilicate minerals, known for their unique tubular structure, which have garnered significant interest for a wide range of applications. This study explores the morphological changes of HNTs when subjected to thermal treatment ranging from 25 °C to 1100 °C using a combination of experimental characterization techniques and molecular dynamics simulations. Techniques such as solid-state NMR (SSNMR), X-ray diffraction (XRD), X-ray photoelectron spectroscopy (XPS), Brunauer–Emmett–Teller (BET) surface area measurements, and Fourier Transform Infrared Spectroscopy (FT-IR) were employed to analyse the structural evolution. The results reveal two major transitions: the first occurring between 400 and 500 °C, corresponding to the release of intercalated water and partial distortion of the HNT structure, and the second occurring between 900 and 1000 °C, marked by the collapse of the tubular structure and the exposure of alumina on the surface. These findings provide significant insights into the thermal stability of HNTs, informing future applications, especially in high-temperature environments.

## Highlights

• Experimental-computational analysis conducted on halloysite calcined from 25 to 1100 °C.
• Atomistic simulations show loss of intercalated water between 400 and 500 °C.
• Atomistic simulations reveal that halloysite structure collapses between 900 and 1000 °C.
• ^29^Si SSNMR shows a shift of silicon peak with increasing calcination temperature.
• ^27^Al SSNMR shows a shift of aluminium peak with increasing calcination temperature.

## Graphical abstract

**Figure:**
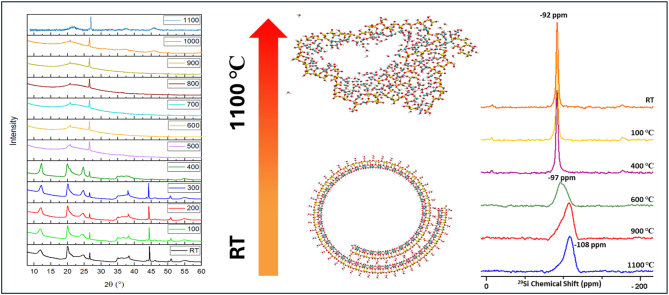

## 1 Introduction

Halloysite nanotubes (HNTs) (Al_2_Si_2_O_5_(OH)_4·_nH_2_O) are aluminosilicate minerals characterised by nanoscale tubular structures, composed of hydrated alumina and silica layers. HNTs are a member of the kaolin group, which also includes the dioctahedral minerals kaolinite, dickite, and nacrite, and the trioctahedral minerals chrysotile, antigorite, chamosite, and cronstedite [1]. HNTs consist of a 1:1 layered aluminosilicate, with an arrangement of one silica tetrahedral sheet (SiO_2_) over an alumina octahedral sheet (Al(OH)_3_), forming a layer with 7 Å thickness. HNTs are considered to be the hydrated form of kaolinite clay due to the presence of interlayer water molecules. The main difference between the structure of HNT and kaolinite is the rolling of layers in HNT, creating its tubular morphology, while kaolinite has a more planar configuration [2,3]. HNT exists in the hydrated form, “halloysite-10 Å”, where a water molecule exists in between the multilayers interlayers, and the dehydrated form, “dehydrated-“halloysite-7 Å”, which lacks the interlayer water molecules due to mild heating or a vacuum environment [4]. As a result, the hydration state of HNTs profoundly impacts their structural integrity and reactivity, further influencing their applications. Another critical feature of halloysite is the presence of surface hydroxyl groups, which play a significant role in surface modification for enhanced functionality. These groups are particularly useful for chemical grafting and the immobilization of active molecules on the surface [3,[5], [6], [7]].

HNT-based materials exhibit remarkable properties and functionalities, making them a subject of intense research across various scientific fields. Over the years, researchers have sought to explore and understand the micromorphological properties of halloysite nanotubes to tailor their functionalities for various practical uses. The unique tubular structure provides a high surface area and porosity, making HNTs excellent candidates for applications in catalysis [8,9], drug delivery, gas separation [10,11], adsorption and environmental remediation [12]. In addition, their exceptional mechanical properties, thermal stability, and biocompatibility expand their potential applicability in nanotechnology and biomedical fields [13].

For example, halloysites have been largely employed in nanotechnology, in combination with other materials, in composites and hybrids, including polymers, carbohydrates, dendrimers, carbon, polydopamine, porphyrin, ionic liquids, hydrogels, and metal-organic frameworks, among others [[14], [15], [16], [17], [18]]. In particular, in polymer nanocomposites, HNTs serve as fillers to enhance the mechanical, thermal, and barrier properties of polymers [19,20]. The hollow structure and high aspect ratio of HNTs contribute to significant improvements in the strength, toughness, and heat resistance of materials such as polypropylene, polystyrene, and polyurethane. Additionally, the incorporation of HNTs into polymer matrices has been shown to increase their barrier properties, making these nanocomposites particularly useful in the packaging industry, especially for food storage, where preventing the permeation of gases and moisture is critical [13]. Notably, the structural integrity of halloysites is generally preserved across the fabrication of these nanocomposites/hybrids, although certain conditions, like carbonization, may lead to partial structural alterations [21].

In the biomedical field, HNTs have extensive applications in controlled drug delivery systems, often in combination with polymers, due to their ability to encapsulate and release therapeutic agents in a sustained and controlled manner [11,[22], [23], [24]]. The tubular structure of halloysite allows drugs or bioactive molecules to be loaded into its hollow lumen, ensuring the release is carefully modulated over time. This property makes halloysite an ideal candidate for targeted drug delivery, where the slow and controlled release of medication can enhance therapeutic outcomes and minimize side effects. Beyond drug delivery, halloysite also shows promise in tissue engineering, particularly in bone regeneration. HNTs can be incorporated into biodegradable scaffolds, providing the necessary structural support for cell growth and tissue formation, while serving as carriers for bioactive molecules that promote healing [[25], [26], [27], [28]].

Environmental applications of halloysite have become a growing area of research [29]. Its high adsorption capacity makes HNTs excellent candidates for water purification systems, where they effectively remove heavy metals, dyes, and other organic pollutants from wastewater. Furthermore, halloysite has been employed in soil remediation efforts, immobilizing harmful substances such as pesticides and fertilizers, thereby preventing environmental contamination and enhancing soil quality [30]. These environmental applications leverage halloysite's large surface area and tunable chemical properties, making it an eco-friendly material for sustainable practices [31].

The inherent physico-chemical properties of halloysite, particularly its tubular morphology, make it a valuable resource for catalytic applications [9,[14], [15], [16]]. Indeed, this unique structure facilitates adsorption and protection of active species, a feature that might even have played a crucial role in the origin of life, a hypothesis yet to be thoroughly investigated [32]. Among the many applications of HNTs, catalysis is a key area where the effect of temperature on the morphology of the support is of crucial importance. As inorganic supports, HNTs prevent sintering of metal nanoparticles, offering solid and durable support [33]. Their large aspect ratio and surface area enhance the local concentration of reactants, benefiting catalytic reactions [[33], [34]]. The use of HNTs not only augments the catalytic activity of loaded species, but also contributes to the development of innovative catalysts for industrial applications, proving cost-effective for green reactions and playing a significant role in processes like cracking of petroleum products, biodiesel production, synthesis of pharmaceutical scaffolds, photocatalysis, green chemistry, biomass conversion, and gas-phase reactions [33,35,36]. In particular, gas-phase reactions play a key role in high-temperature industrial processes such as methane reforming, Fischer-Tropsch synthesis, catalytic cracking, CO₂ methanation, and hydrogenation.

In this regard, HNTs have been reported as effective supports for metal catalysts like nickel (Ni), lanthanum (La), and cobalt (Co), which are commonly used in dry methane reforming to produce synthesis gas [9,36,37]. The use of halloysite helps mitigate catalyst deactivation caused by carbon deposition (coking), a common issue in methane reforming reactions. By dispersing the active metal sites and providing better resistance to sintering, halloysite supports enable more sustainable and efficient methane reforming processes. These systems have demonstrated good catalytic conversion and stability within the typical reaction temperature range of 700–850 °C. In the field of Fischer-Tropsch synthesis, a process that converts syngas into hydrocarbons, halloysite-supported cobalt (Co) and ruthenium (Ru) catalysts have shown potential in enhancing the selectivity and conversion efficiency of syngas into liquid fuels, with reaction temperatures ranging from 250 to 450 °C [[38], [39], [40]]. The tubular morphology of halloysite facilitates better contact between the reactant gases and the active catalytic sites, leading to improved performance in gas conversion processes. Halloysite nanotubes are also being explored for their applications in catalytic cracking, where large hydrocarbon molecules are broken down into smaller, more valuable compounds like gasoline, olefins, and other light hydrocarbons. One of the key advantages of using halloysite in catalytic cracking is its thermal and mechanical stability, which allows it to withstand the harsh conditions of the cracking process, with reaction temperatures often exceeding 600 °C. The catalytic cracking process is crucial in refining crude oil, and the unique properties of halloysite make it a promising material for enhancing this process [[41], [42], [43]]. In the context of addressing climate change, halloysite-supported catalysts are being explored for the conversion of carbon dioxide (CO₂) into useful chemicals or fuels [44,45]. For example, in the Sabatier reaction, CO₂ is hydrogenated to produce methane (CH₄) and water at temperatures between 300 and 500 °C. Halloysite nanotubes serve as ideal supports for nickel-based catalysts, which are commonly used in the methanation process. As seen in other catalytic applications, the high surface area and chemical stability of halloysite enhance the dispersion of the active metal sites, improving both the efficiency and selectivity of the CO₂ conversion reaction. Finally, halloysite nanotubes can serve as effective support in hydrogenation reactions. In hydrogenation, unsaturated compounds such as alkenes and alkynes are converted into saturated compounds through the addition of hydrogen. The tubular structure of halloysite provides a high surface area for metal nanoparticle dispersion, preventing agglomeration and enhancing the efficiency of the catalytic reaction. For instance, palladium-supported halloysite catalysts have been shown to facilitate hydrogenation reactions with high selectivity and conversion rates, making them suitable for applications in fine chemicals and petrochemical industries [[46], [47], [48]].

The diverse range of chemical transformations facilitated by these halloysite-based systems, ranging from hydrogenation and oxidation to coupling reactions and photocatalytic degradation, highlights their versatility and effectiveness even at high temperatures.

A small number of studies over the past decades have been dedicated to the exploration of the thermal transformation of HNT [[49], [50], [51], [52], [53]]. About three decades ago, Smith et al. conducted a comprehensive study investigating the thermal decomposition of HNT, by solid-state NMR, under gradual heating up to 1400 °C. They concluded that the thermal transition of HNT was very similar to that of kaolinite [52]. Yuan et al., on the other hand, provided evidence of morphological changes in HNT when heated to 1100 °C, such as the closing of the nanotube at one or both terminal ends. They also observed that HNTs maintained their overall tubular morphology and mesoporosity at heating temperatures below 900 °C. The differences in the studies about the thermal transformation of HNT under high temperature heating is related to the impurities present in many HNT samples, such as quartz and cristobalite. Hence, information pertaining to the HNT purity and heating conditions is vital [54]. More recently, Asgar et al. investigated the structural and microstructural evolution of halloysite and kaolinite upon thermal treatment. The study highlights how different morphologies (tubular for halloysite and planar for kaolinite) affect their thermal behaviors, using advanced X-ray scattering techniques to explore changes in pore structure and surface roughness [49]. Whereas the group of Santagata meticulously explored the behavior of nanoconfined water within halloysite: their work discusses how water confined in the lumen and interlayer spaces behaves under various thermal conditions and freezing temperatures, using thermal analysis and differential scanning calorimetry to examine dehydration processes [55].

When using halloysite clay in high-temperature applications, such as catalysis, it is crucial to understand the structural, morphological, and chemical changes that occur with heat. While some investigations have delved into the structural changes of HNTs at elevated temperatures, there lies a notable gap in correlating the effect of heating on the macroscopic structure of halloysite with the corresponding changes at atomistic scale. To address these critical inquiries, we herein explore the morphological changes of HNT in the room temperature (RT) to 1100 °C range, by employing a comprehensive approach that combines several experimental characterization techniques and molecular dynamics (MD) simulations. Specifically, solid-state NMR (SSNMR) is utilized to provide valuable insights into the composition, bonding, and arrangement of silicon and aluminum atoms within the HNT clay structure. Simultaneously, structural changes have been studied with X-ray powder diffraction (XRD) as well as X-ray photoelectron spectroscopy (XPS) measurements. Furthermore, BET surface area measurements are conducted, providing a comprehensive portrayal of the surface area and pore size distribution as a function of temperature. Additionally, Fourier Transform Infrared Spectroscopy (FT-IR) measurements are employed to assess changes in chemical bonds. The molecular dynamics (MD) simulation study is conducted to identify the structural changes of HNT with rising temperatures. as a promising method to investigate properties of the structure of HNT at the atomic level. This integrated methodology enables the probing of features across spatial scales ranging from angstroms to macroscale measurements, which facilitates the evaluation of the structural metamorphosis of halloysite sheets transitions from crystalline to amorphous phases.

Our atomistic simulations reveal that the first transition (i.e., between 400 and 500 °C) corresponds to a distortion of the HNT structure and the release of intercalated water between the HNT layers, whereas during the second transition (i.e., between 900 and 1000 °C) the HNT structure collapses and alumina is exposed on the surface of the HNT. These transitions have been consistently observed in all our experimental results. This multifaceted study provides strong evidence to significantly advance the understanding of the structural and morphological evolution of the tubular hierarchically ordered aluminosilicate HNT, shedding light on its behaviour at different thermal stages, enriching the knowledge base for future synthesis, modification techniques, as well as thermal applications.

## 2 Materials and methods

Halloysite nanoclay (kaolin clay), Al_2_Si_2_O_5_(OH)_4_·2H_2_O, obtained from Sigma Aldrich (made in USA) was subjected to thermal treatment in a muffle furnace. Individual HNT samples were heated for 1 h from 100 °C up to 1100 °C, in 100 °C intervals, to assess the morphological alterations.

Solid-state NMR measurements were performed at a magnetic field strength of 14.1 T on a Bruker AVANCE III wide bore 600 MHz spectrometer. The experiments were conducted at Larmor frequencies of 119.2 MHz for ^29^Si and 156.4 MHz for ^27^Al, with the spectrometer equipped with a 3.2-mm triple-resonance low-temperature magic angle spinning (LT-MAS) NMR probe from Bruker. The samples were finely ground and packed into zirconia rotors with an outer diameter of 3.2 mm for analysis. The ^29^Si NMR spectra were collected at 10 kHz MAS rate using rotor-synchronized Hahn spin-echo pulse sequence (π/2-*τ*-π-*τ*-ACQ) [56,57] with a *τ* value of 100 μs, a π/2 excitation pulse of 4.5 μs, and a recycle delay of 5 s for the accumulation of 1280 transients. All ^29^Si NMR chemical shifts were externally referenced to liquid tetramethylsilane (TMS) set at 0 ppm. The ^27^Al NMR spectra were collected at 12.5 kHz MAS rate using rotor-synchronized Hahn spin-echo pulse sequence (π/2-*τ*-π-*τ*-ACQ) with a *τ* value of 80 μs, a solid π/2 excitation pulse of 1.6 μs, and a recycle delay of 3s for the accumulation of 1024 transients. Additionally, ^27^Al single-pulse (SP) experiments were performed on some samples at 12.5 kHz MAS rate using a small ^27^Al excitation pulse of 0.6 μs (π/15 flip angle) and a 3s recycle delay with the accumulation of 128 transients. All ^27^Al NMR chemical shifts were externally referenced to a 1.0 mol/L Al(NO_3_)_3_ aqueous solution set at 0 ppm.

FT-IR spectra of the HNT samples were obtained in transmittance mode using a Thermo Scientific Nicolet iS50 FT-IR spectrometer, equipped with an attenuated total reflectance (ATR) accessory featuring a diamond crystal plate. The spectra were collected over 64 scans per sample and background, covering the spectral range of 4000–400 cm⁻^1^ with a resolution of 4 cm⁻^1^.

The specific surface areas of the synthesized catalysts were determined using a Micromeritics ASAP 2420 surface area analyzer at 77 K. Prior to analysis, samples were dried at 90 °C for 30 min and degassed under vacuum at the same temperature for 6 h to eliminate contaminants and moisture. The specific surface area was calculated using the BET equation, while pore size analysis was conducted using BJH desorption.

X-ray diffraction (XRD) patterns of the catalysts were recorded using a Shimadzu XRD-6100x diffractometer, employing Cu-Kα radiation (λ = 1.5406 Å). The instrument was calibrated to operate at 30 mA and 40 kV, with a scanning speed of 7°/min over a 2θ range of 10°–70°.

Transmission electron microscopy (TEM) images were captured with a JEOL 2010 transmission electron microscope operating at 200 kV. Samples were prepared by dispersing the materials in ethanol, then depositing a drop of the suspension onto a thin copper grid. Once the ethanol evaporated, the grid was inserted into the microscope for imaging.

The XPS measurement was conducted on a Thermo Fisher EXCALAB250 platform with a monochromatic Al K2 x-ray source of 1486.8 eV and a hemisphere analyzer. The total energy resolution was better than 0.5 eV. All measurements were conducted under ultrahigh vacuum (under 10^−9^ mbar) at room temperature. The atomic ratio of different elements was estimated by calculating the area of the corresponding high-resolution at a pass energy of 20 eV. The binding energy was calibrated with the C1s at 284.8 eV.

Molecular dynamics (MD) at semi-empirical tight binding (GFN-xTB) level [58] with Grimme dispersion correction (D3) [59] were performed to obtain molecular insights about the Al halloysite HNT thermal stability. The initial HNT structure was taken from Ref. [60]: a spiral halloysite already optimized of 1 nm winding axis. The structure was centred in a box of 20 × 20 × 0.52 nm^3^ X, Y, Z dimension, respectively, and full periodic boundary conditions, resulting in a long HNT spiral nanotube along the Z-direction. The HNT was solvated with one water layer, resulting in a water intercalated layer between the two halloysite sheets and on the outer part of the HNT exposed to the gas phase. The total number of atoms in the simulation box was 1380. The structure was further related at xTB level. Starting from the relaxed geometry, we performed six independent MD simulations at constant volume and temperature (i.e., NVT ensemble) at 25 °C, 100 °C, 400 °C, 500 °C, 800 °C, and 1000 °C. The time step was set to 0.5 fs. The temperature was kept at the desired value by using a Nose-Hoover thermostat (barostate) with a constant of 50 fs [61]. Similar computational set-up has been used in literature to study weakly hydrated HNT [62]. All simulations were performed using CP2K [63] molecular dynamics package.

## 3 Results and discussion

### 3.1 Morphology and physicochemical properties

**Fig. 1:**
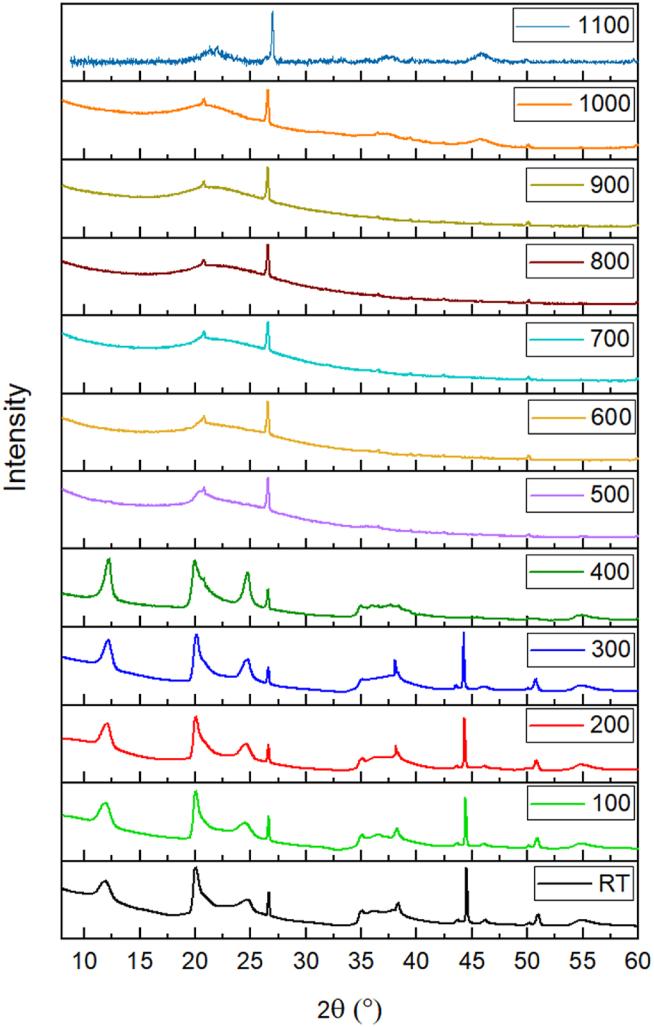
XRD patterns of the calcined HNT samples at different temperatures.

From the X-ray diffraction (XRD) profiles of pure HNT it was confirmed that the high temperatures can induce phase transformations in halloysite nanotubes, leading to changes in their crystal structure and composition. Fig. 1 presents a sequential phase transformation process of HNT at varied calcination temperatures, where it undergoes an initial transition to a 7 Å-type crystal structure with basal (001) reflection at ∼12.05° 2θ prior to reaching 400 °C, followed by a transformation into an amorphous state within the temperature range of 500–1000 °C, which is also manifested in the ^29^Si MAS NMR spectra shown below in Fig. 6. The peak intensity increased with increasing temperature from room temperature to 400 °C, and the full width at half maximum (FWHM) decreased. This is due to the dehydration process that involves the removal of physically bound or adsorbed water molecules from the surface and interlayer spaces of HNT, resulting in a decrease of interlayer spacing and a shift to lower angle in diffraction peak [64,65]. It is worthy to note here that some peaks are still being observed with increasing the temperature from room temperature to 400 °C. This is because of the tubular structure in halloysite that conserves the stability of pseudomorphic aggregation in tetrahedral sheet to certain temperatures extent [66].

Moreover, it can be noticed from the XRD patterns that the collapse of the octahedral layer in halloysite resulted in a rapid decrease in diffraction intensity as temperature increased. This phenomenon was primarily attributed to the release of structural water and escape of large amounts of hydroxyls from the lattice. The significant loss of structural water occurred predominantly at 500 °C. Between temperatures of 500–900 °C, the patterns showed broad diffraction maxima owing to the process of dehydroxylation that led to the gradual separation of silica and alumina, which were initially located in the tetrahedral and octahedral sheets, respectively. This separation caused a disruption in the long-range order [54], which is also confirmed by the broad line shapes observed in the ^29^Si MAS NMR spectra of halloysites at 600 °C and beyond in Fig. 6. However, a wide reflection emerged at 1000 °C, indicating the collapse of the tubular structure as verified by the molecular dynamic's observation shown in Fig. S4. This is attributed to the reduction in crystallite size and an increase in lattice defects [65], as well as to the presence of dissociated amorphous SiO_2_ of mullite and cristobalite separated from the metahalloysite. It could also be attributed to the existence of nanocrystalline γ-Al_2_O_3_, as indicated by the new formed diffraction peaks at 2θ = 37.5 °C and 45.8 °C for 1000 °C and 1100 °C- based samples, as previously observed by Yuan et al. and other authors, and as confirmed by ^27^Al MAS NMR analysis in Fig. S3 [53,54,64].

**Fig. 2:**
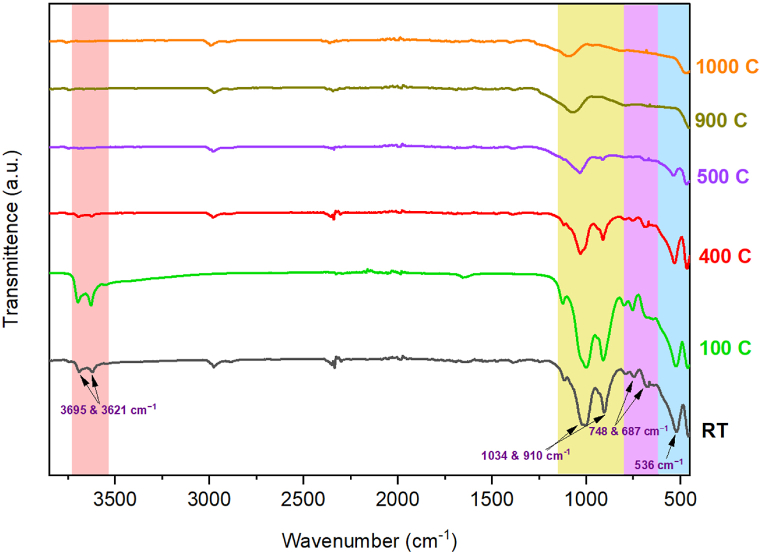
FT-IR spectra of the six HNT samples corresponding to the temperatures of the most relevant transitions, stretching vibration of Al-OH (red), O–Si–O and Al–O–OH (yellow), apical Si–O (purple), and Al–O–S (blue). (For interpretation of the references to color in this figure legend, the reader is referred to the Web version of this article.)

FT-IR analysis was conducted to examine changes in the vibrational spectra of HNTs as a function of temperature (Fig. 2 and Fig. S1). The peaks of the HNT-RT (Fig. 2), which appear at 3621 and 3695 cm^−1^, are the stretching vibration of the inner surface of Al-OH groups [55], while the band at 536 cm^−1^ indicates the deformation vibration of Al–O–S. Highly intense absorption peaks corresponding to O–Si–O and Al–O–OH vibrations were detected around 1034 cm^−1^ and 910 cm^−1^, respectively, while the peaks at 687 and 748 cm^−1^ are attributed to the stretching mode of apical Si–O [67,68].

At lower temperatures (e.g., 100 °C–300 °C), the FT-IR spectra in Fig. 2 show prominent peaks associated with hydroxyl (OH) stretching vibrations, indicating the presence of water molecules and hydroxyl groups in the HNTs. However, as the temperature rises towards 500 °C–900 °C, these hydroxyls (OH) stretching bands gradually decrease in intensity or shift in position due to dehydration and dehydroxylation processes, in good agreement with the XRD patterns and the other utilized techniques. At higher temperatures (e.g., 1000 °C and 1100 °C), changes in the FT-IR spectra indicate phase transformations or structural transitions in the HNTs. New peaks or shifts in peak positions may emerge, reflecting alterations in the crystalline phases or the formation of metastable phases as indicated in the XRD and SSNMR results. These changes provide insights into the thermal stability and transformation pathways of the HNTs under extreme temperature conditions [51].

**Fig. 3:**
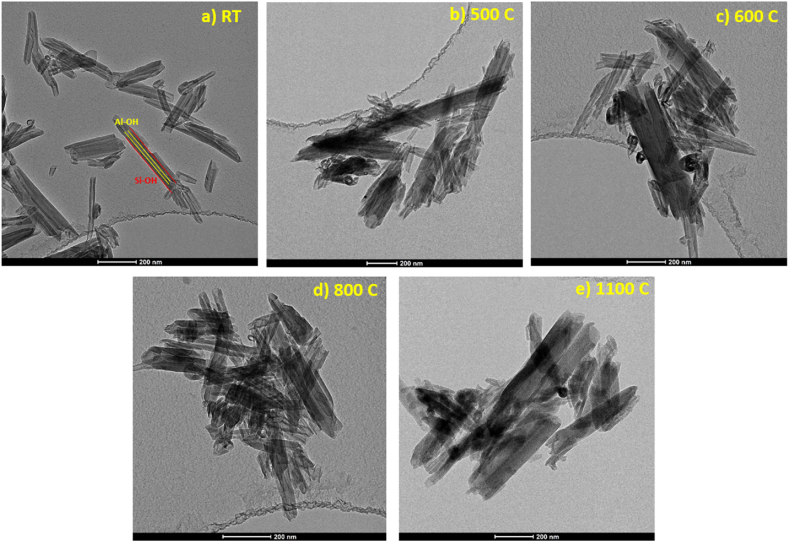
TEM images of the five representative HNT samples at RT, 500, 600, 800, and 1100 °C.

TEM images of halloysite revealed that the untreated halloysite particles (Fig. 3a) exhibited characteristic cylindrical forms with transparent central regions extending longitudinally along the length of the cylinder. These cylindrical forms indicate the silanol and aluminol groups on the nanotubes' exterior and internal surfaces, respectively. However, at elevated temperatures 600–1100 °C (Fig. 3c and d and e), clusters or bundles of HNTs were observed, indicating the propensity for aggregation and the influence of thermal energy on interparticle interactions where the mobility of HNTs is increased, resulting in the fusion of adjacent nanotubes and the formation of larger agglomerates. It is also worth noting that, while the tubular morphology of HNT remained intact at these increased temperatures except for 1100 °C, surface mottling and deformed tubular outlines were detected, indicating a significant degree of structural disorder and a special degradation of the inner Al layer. All these observations are most likely due to the structural disordering related to dehydroxylation and the collapsing of the inner lumen, based on the findings of molecular dynamics, XRD, and SSNMR analyses. Similar structural changes have been observed by Wu et al. [65].

**Fig. 4:**
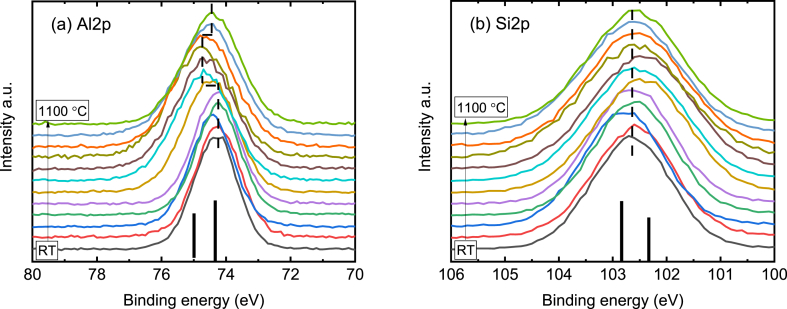
The evolution of Al2p and Si2p as a function of temperature.

**Fig. 5:**
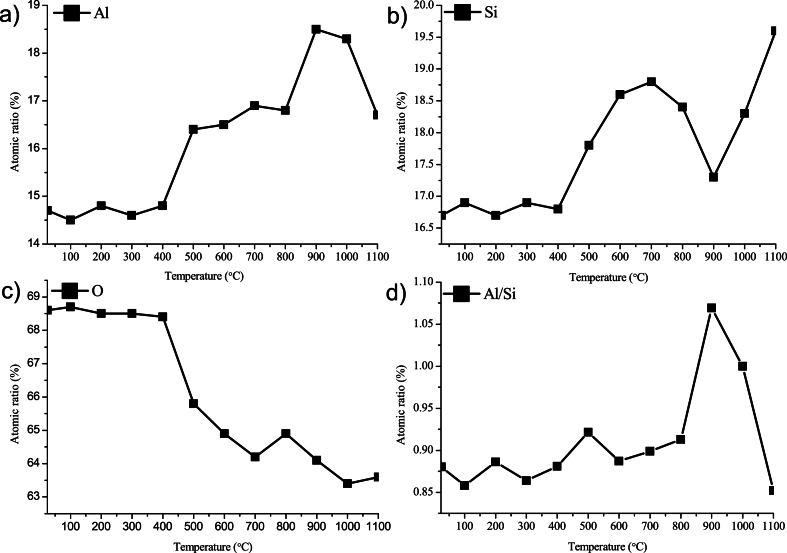
The atomic ratio of Aluminum (a), Silicon (b), Oxygen (c) and the Al/Si (d), respectively.

**Fig. 6:**
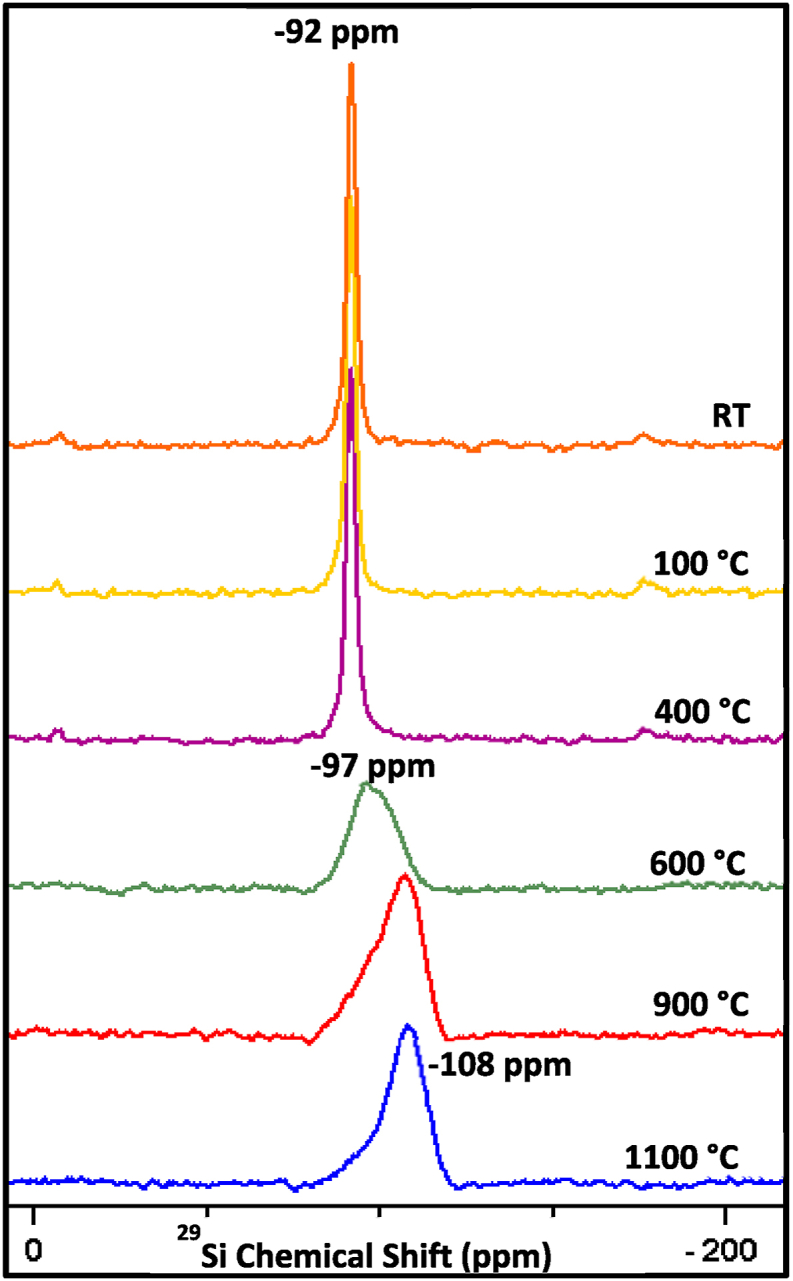
Solid-state ^29^Si NMR spectra at 10 kHz MAS rate of the calcined HNT samples at various temperatures.

**Fig. 7:**
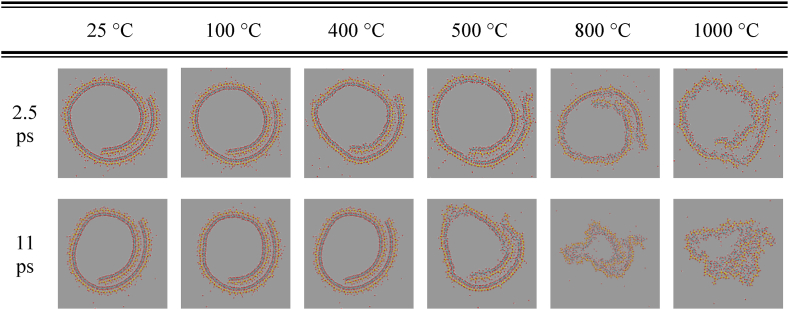
View of the HNT structures from the cylindrical axis at different temperatures, at 2.5 ps (top panels) and 11 ps (lower panels). Atom color code: Al (cyan), Si (yellow), O(red), and H (white). (For interpretation of the references to color in this figure legend, the reader is referred to the Web version of this article.)

The evolution of the Al2p and Si2p as a function of temperature is shown in Fig. 4 (a) and (b) respectively. The Al2p in Fig. 4(a) shows clear energy shift above 500 °C, which indicates a bond formation of Al-O; the two black bars suggest the possible aluminosilicate at 74.4 eV and Al oxidation at 75.0 eV. Meanwhile, the Si2p in Fig. 4(b) gives less shift but at high temperature a broadening is still visible, with a possible silicate component at 102.5 eV and an additional peak at higher binding energy side related to the silicon oxidation. The variation of atomic concentration of Al, Si and O are given in Fig. 5, Fig. 6, which are carefully calculated from the area of the Al2p, Si2p and O1s high-resolution spectra. As shown in Fig. 5(a–c), at temperature below 500 °C, no variation is observed for all 3 elements. The simultaneous intensity enhancement of both Al and Si at 500 – 600 °C may originate from the depletion of oxygen content (Fig. 5(c)), which is confirmed by the fixed Al/Si ratio in Fig. 5(d) below 800 °C. An abrupt variation of the Al/Si ratio is given at 900 °C, which increases to 1.07 from an average value of 0.9 at lower temperature, but then decreases dramatically to 0.85 with the temperature further increasing to 1100 °C. Our MD simulation in Fig. 7 has indicated a structural collapse at temperatures above 800 °C with ruptures of the silica layer, which may lead to significant number of Al atoms exposed to the X-ray beam, which contributes to the increase of Al/Si ratio in Fig. 5(d). As the temperature increases and the HNT further collapses on itself, the silica layers bend and collide, covering the Al layer and supressing the Al exposure.

Solid-state nuclear magnetic resonance (SSNMR) spectroscopy was employed to investigate the effects of calcination temperature on the silica outer layer of HNT, and the resulting ^29^Si and ^27^Al MAS NMR spectra are shown in Fig. 6 and Fig. S2, respectively. The obtained results indicate that heat treatment up to 400 °C does not cause any noticeable change in the silicon local electronic environment of the pristine HNT, hence no changes in the HNT crystalline structure. The ^29^Si MAS NMR spectra of HNT, treated at 100 °C and 400 °C, each shows a sharp silicon signal at −92 ppm from TMS, identical to that of the pristine HNT. This peak could be assigned to a single Q^3^-type Si(OSi)_3_(OAl_2_) silicon site, suggesting that the HNTs retain their crystalline structure up to this point [69,70]. However, as annealing temperature is increased to 600 °C, a new broad featureless peak is observed at −97 ppm, indicating an amorphous silicon environment [52,71,72]. This shift suggests a loss of order in the nanotube walls and a reduction in crystallinity, denoted by the silicon local environment becoming less structured and more random. As the temperature is increased to 900 °C, the peak shifts to −108 ppm, corresponding to Q^4^-type Si(OSi)_4_ amorphous silica-like sites [9,73,74], which implies a significant transformation of the silicon structure within the HNTs and the complete loss of the original crystalline nanotube framework. The ^29^Si MAS NMR spectrum of the HNT treated at 1100 °C shows similar peak to that of the HNT at 900 °C, reinforcing the conclusion that the silicon environment remains amorphous at this elevated temperature and that no further structural changes occur beyond the transition noted at 900 °C. Of note is the agreement between the experimental MAS NMR findings and those obtained from MD simulations discussed below, as well as the XRD results discussed above. This consistency at high temperatures suggests the stability of the amorphous silicon environment once formed. This progressive transformation from a well-defined crystalline state to an amorphous state with increasing calcination temperature provides a valuable understanding of the thermal stability and structural evolution of halloysite nanotubes, which is crucial for potential applications that require thermal processing.

To elucidate the effects of thermal treatment on the structure/order of the alumina sheets in HNT, the calcined samples were further investigated by solid-state ^27^Al NMR spectroscopy under magic-angle-spinning (MAS) conditions, and the obtained spectra are shown in Figure S2 and Figures S3. The ^27^Al MAS NMR spectrum shows a single sharp peak centred at 5.3–5.5 ppm up to 400 °C, as shown in Fig. S2. This peak corresponds to an octahedrally coordinated aluminium site (AlO_6_) [52,75]. By 600 °C, however, substantial changes are observed, as indicated by the appearance of two new broad peaks centred at 51 and 28 ppm along with a residual peak at 3.3 ppm. These three distinct ^27^Al peaks could be assigned to a tetrahedrally coordinated AlO_4_ site centred at 51 ppm, a penta-coordinated AlO_5_ site centred at 28 ppm, and a residual octahedral AlO_6_ site at 3.3 ppm [[76], [77], [78]]. It is well known that the ^27^Al NMR parameters depend strongly on the coordination number and local geometry around the Al centre [52,79] and that the ^27^Al NMR chemical shift ranges of AlO_x_ sites in aluminosilicates and other systems are well established in the literature [52,80]. The changes observed in the ^27^Al spectrum upon dehydroxylation should not be surprising, as the hydroxyl groups (-OH) are closely coupled to the alumina layer of the HNT framework. Moreover, penta-coordinated AlO_5_ phases have been reported to be more common in disordered aluminosilicates and related systems than in crystalline systems [52,80,81]. Both ^29^Si and ^27^Al NMR spectra presented here evidently reveal that the initial dehydroxylation stage, starting above 400 °C, results in a substantial loss in the crystallographic order within the HNT framework, which is manifested by the considerable broadening of the observed lineshapes in both sets of spectra. The ^27^A1 spectrum remains mostly unaffected up to 1000 °C, where only two distinct peaks centred at 9.5 ppm (AlO_6_) and 63 ppm (AlO_4_) are observed with a noticeable narrowing in lineshapes, which is a clear indication of nanocrystalline γ-Al_2_O_3_ as revealed by XRD results discussed above. As shown in Fig. S3, the ^27^Al MAS NMR spectrum of HNT calcined at 1000 °C is consistent with γ-Al_2_O_3_ whose ^27^Al NMR spectrum was acquired under identical conditions. At 1100 °C, the ^27^Al NMR spectrum reveals a modest but definite peak centred at 40 ppm, in addition to the octahedral peak at 9 ppm and the tetrahedral peak at 64 ppm. The new peak can be attributed to a tetrahedral AlO_4_ phase, an indication of the plausible onset of a mullite-like phase [76,82]. The lineshapes of the ^27^Al MAS NMR spectra in Figs. S2 and S3 match well with those previously reported by Smith et al. [52].

In conclusion, the ^29^Si and ^27^Al MAS NMR results presented here collectively reveal that the initial dehydroxylation stage (400–500 °C) leads to a decrease in the long-range crystalline order within the silica and alumina layers, caused by a gradual disconnection of the two layers. However, the alumina and silica layers respond with different mechanisms to the second dehydroxylation step (900–1000 °C), where the alumina layer is more profoundly affected through the formation of crystalline γ-Al_2_O_3_ followed by the beginning of mullite-rich phase formation upon further heating.

**Fig. 8:**
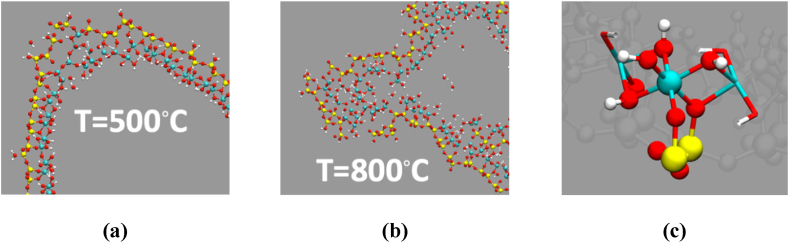
Panel (a) and (b), zoom of HNT structures from Fig. 7 at 11 ps at 500 °C and 800 °C, respectively. Panel c) a zoom on the structure showing the coordination of Al with 4 hydroxyl group and two -O-Si-. Atom color code Al (cyan), Si (yellow), O(red), and H (white). (For interpretation of the references to color in this figure legend, the reader is referred to the Web version of this article.)

Silicon and aluminum atoms in the tetrahedral and octahedral layers of HNTs, respectively, are associated with hydroxyl groups. Dehydroxylation involves the breaking of Si-OH and Al-OH bonds, resulting in the removal of hydroxyl groups from the crystal lattice. Dehydration and dehydroxylation processes can lead to structural rearrangements and alterations in the coordination environment of Si and Al atoms, impacting the stability and properties of HNTs. In this regard, Fig. 7 shows the HNT spiral structure at different temperatures after 2.5 ps and 11 ps MD simulation. The HNT structure is stable over the 11 ps of the MD up to 400 °C. The drastic change occurs at 500 °C, where some important structural changes were observed, such as the breaking of the outer Si layer (see Fig. 8(a)). Interestingly, the intercalated water layer between the two HNT sheets still survives at 500 °C after 11 ps of MD simulations. However, at higher temperatures (i.e., 800 °C and 1000 °C), the intercalated water layer evaporates, and we observe a larger fraction of break points in the Si outer layers and the formation of Al-oxides (Fig. 8(b)). Between 800 °C and 1000 °C the tubular structure of HNT collapses. To summarize, in the timescale of tens of ps, MD simulations show stable HNT structure and intercalated water layer up to 400 °C.

**Fig. 9:**
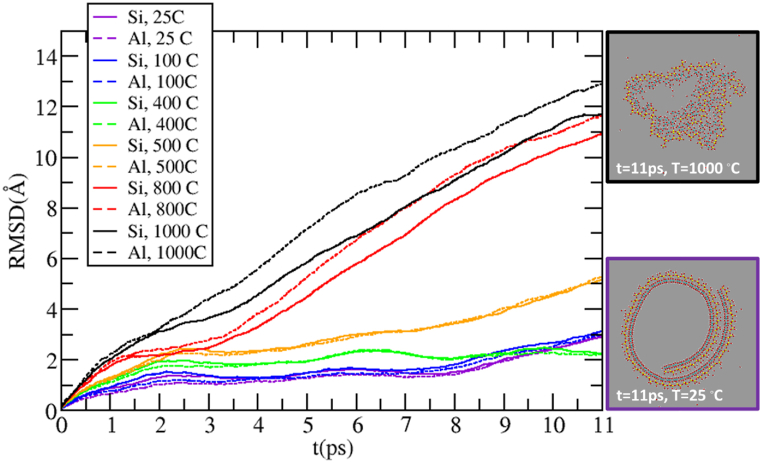
The root mean square displacement for the Si and Al atom coordinates from the initial conformation as a function of time in the HNT structures at different temperatures.

A more quantitative description of the structural change over time can be obtained by recording the root mean squared displacement (RMSD) from the initial starting structure for the Al and Si atoms of the HNT (Fig. 9). RMSD suggests, as qualitatively inferred from snapshots in Fig. 8, Fig. 9, that the structure undergoes a mild rearrangement at 500 °C, and a larger one at 800 °C and 1000 °C. For all the investigated temperatures, the Al RMSD is larger than that of Si, indicating that the structural re-arrangement is more important for the inner Al layer than for the outer Si one: this difference between Al and Si RMSD is particularly evident at 800 °C and 1000 °C. Even if longer simulations might be needed, this initial observation suggests that the inner layer of the HNT is more subject to degradation with temperature than the outer one. Our MD simulation results (Fig. 7, Fig. 8, Fig. 9) qualitatively and quantitatively identify two transition temperatures. A first transition, between 400 and 500 °C, corresponds to a distortion of the HNT structure and the release of intercalated water between the HNT layers; and a second transition between 900 and 1000 °C shows structure collapses and exposure of alumina on the HNT surface. The exposure of the alumina to the surface of the HNT becomes even more evident at higher temperatures (e.g., 1100 °C Fig. S4). These temperature transitions have also been consistently observed in all our experimental results reported above.

The simulations here discussed represent an evolution of those previously reported by our group based on molecular dynamics only at 300 and 800 K obtained after ∼10 ps [62].

### 3.2 Textural properties

Table 1 presents the variation of average pore diameter, micropore volume, Langmuir surface area, total pore volume, micropore area, and surface area of the halloysite nanotube samples subjected to thermal treatment from room temperature up to 1100 °C. The high temperature can affect the pore structure of halloysite nanotubes by altering their size, shape, and distribution. Dehydration and dehydroxylation processes that involve breaking of the hydroxyl (OH) bonds within the crystal lattice of HNTs, may lead to the collapse of internal pores or the formation of new pores, affecting the surface area and porosity of the nanotubes.

**Table 1:** Surface Area & Pore Size Analysis of HNT at different calcination temperatures.

Sample	BET Surface Area, S_BET_ [m^2^/g]	Langmuir surface area [m^2^/g]	Micropore area [m^2^/g]	Micropore Volume [cm^3^/g]	Total Pore Volume, V_t_ [cm^3^/g]	Average pore width [nm]
25 °C	50.33	64.89	10.11	0.004490	0.242788	14.72
100 °C	54.02	60.72	4.17	0.001944	0.254793	16.42
200 °C	51.56	66.20	4.82	0.002272	0.241734	14.07
300 °C	51.06	73.27	5.83	0.002749	0.242897	14.32
400 °C	49.23	72.18	8.33	0.003724	0.248570	14.77
500 °C	48.56	55.09	6.65	0.003044	0.285048	18.50
600 °C	48.81	60.48	7.79	0.003649	0.327927	15.98
700 °C	50.65	59.72	5.57	0.002442	0.140678	8.61
800 °C	49.42	60.06	4.59	0.001998	0.287993	15.09
900 °C	36.32	41.38	4.12	0.001770	0.221645	14.91
1000 °C	30.23	34.79	6.10	0.002609	0.225241	16.73
1100 °C	26.03	29.94	4.44	0.001886	0.223041	17.40

BET surface area (S_BET_) initially increases slightly as the temperature rises from 25 °C to 100 °C, indicating that minor structural changes or removal of contaminants and adsorbed molecules enhance the surface area accessible for nitrogen adsorption. However, beyond 100 °C, a gradual decrease in the BET surface area (in the range of 48–51 m^2^/g) is observed as the temperature increases up to 800 °C, suggesting that the tubular structure is still maintained, followed by a more significant drop at temperatures above 900 °C. This trend suggests that structural collapse or sintering of the nanotubes occurs at higher temperatures, reducing the surface area. The Langmuir surface area begins at 65 m^2^/g, decreases to 60 m^2^/g near 100 °C, peaks at 73 m^2^/g around 300 °C, and then sharply decreases to 30 m^2^/g at 1100 °C following the micropore volume distribution. This could imply changes in the adsorption characteristics of the nanotubes, possibly due to changes in the chemical composition or the development of new surface features that favor monolayer adsorption (γ-alumina). A similar trend has been reported by Wu et al. with a stable BET surface area up to 800 °C [65].

The micropore area and volume generally decrease with increasing calcination temperature, indicating that the microporous structure of the halloysite nanotubes is compromised. The creation and/or enlargement of pores at lower temperatures could be followed by the collapse of these microporous structures at higher temperatures. The total pore volume and average pore width show varied trends across the temperature range. The total pore volume slightly decreases up to 400 °C, increases significantly at 500 °C and peaks at 600 °C, indicating the formation or enlargement of pores. A drastic reduction in pore volume is observed at 700 °C, whereas the S_BET_ was not largely affected. This could be due to the initiation of the collapse of the tubular structure as confirmed by the MD simulation in Fig. S4, a similar observation was reported by Khelifa et al. [50]. According to Fu et al. [83], when the internal channel within the lumen space is not fully blocked, the surface area increases, but the pore volume decreases. This is attributed to the N_2_ gas not being absorbed on the surface of the collapsed layer within the HNT lumen.

The average pore width increases, particularly notable at 500 °C and above, suggesting that the calcination process leads to the development of larger pores, possibly due to the breakdown of smaller pore structures and the fusion of pores. These changes indicate that thermal treatment significantly impacts the pore structure and surface properties of HNTs, which is crucial for their thermal stability and potential applications.

## 4 Conclusion

This study provides a comprehensive analysis of the thermal stability and structural evolution of halloysite nanotubes (HNTs) under a range of temperatures (RT to 1100 °C). Employing a combination of experimental techniques and molecular dynamics simulations, we observed two distinct structural transitions: the first between 400 and 500 °C, and the second between 900 and 1000 °C. These transitions are marked by the transformation of HNTs from an ordered crystalline state to an amorphous arrangement, driven by the gradual collapse of the tubular structure. While the HNT structure is stable up to 400 °C, the HNT cylindrical structure is distorted between 400 and 500 °C (first transition) and evaporation of intercalated water starts. Between 900 and 1000 °C, the HNT structure collapses and alumina is exposed to the surface of the HNT. These structural changes were consistently observed across multiple experimental methods, including SSNMR, XRD, and FT-IR, as well as depicted through computational simulations, offering a robust understanding of the thermal behaviour of HNTs.

This study highlights the importance of monitoring the thermal treatment conditions to preserve the structural integrity of HNTs for various applications. The substantial changes in surface area, pore size distribution, and chemical bonding with increasing temperatures underline the critical role that thermal stability plays in determining the functional performance of HNTs. These findings lay the groundwork for further research into optimizing the synthesis and thermal processing of HNTs for a variety of high-temperature industrial applications. Future work could explore the impact of these thermal transformations on specific applications, advancing the utility of HNTs in fields ranging from energy storage to chemical catalysis.

## CRediT authorship contribution statement

**Ahmed Abotaleb:** Writing – original draft, Visualization, Investigation, Formal analysis. **Ivan Gladich:** Writing – original draft, Visualization, Software. **Kamal Mroue:** Writing – original draft, Visualization, Investigation, Formal analysis. **Nada Abounahia:** Writing – original draft. **Alaa Alkhateeb:** Investigation. **Abdulaziz Al-Shammari:** Formal analysis. **Yongfeng Tong:** Writing – original draft, Formal analysis. **Dema Al-Masri:** Writing – original draft. **Alessandro Sinopoli:** Writing – original draft, Visualization, Supervision, Investigation, Formal analysis, Conceptualization.

## Declaration of competing interest

The authors declare that they have no known competing financial interests or personal relationships that could have appeared to influence the work reported in this paper.
