# Supplementary material for: Impact of thermal treatment on halloysite nanotubes: A combined experimental-computational approach
Source: Heliyon. 2024 Oct 30;10(21):e39952. doi: 10.1016/j.heliyon.2024.e39952 (PMC11565379; doi:10.1016/j.heliyon.2024.e39952)
Supplement: Multimedia component 1 [file mmc1.docx]

**Supporting information**

**Impact of Thermal Treatment on Halloysite Nanotubes: A Combined Experimental-Computational Approach**

Ahmed Abotaleb^1ǂ^, Ivan Gladich^1ǂ^, Kamal Mroue^2ǂ^, Nada Abounahia^1^, Alaa Alkhateb^1^, Dema Al-Masri^3^, Abdulaziz Al-Shammari^2^, Yongfeng Tong^2^, Alessandro Sinopoli^1*^

*^1^* *Qatar Environment and Energy Research Institute, Hamad Bin Khalifa University, P.O. Box 34110, Doha, Qatar*

*^2^ HBKU Core Laboratories, Hamad Bin Khalifa University, P.O. Box 34110, Doha, Qatar*

*^3^ Earthna Center for a Sustainable Future, Qatar Foundation, Doha, Qatar*

*^ǂ^These authors contributed equally to this work.*

*^*^E-mail:* [*asinopoli@hbku.edu.qa*](mailto:asinopoli@hbku.edu.qa)


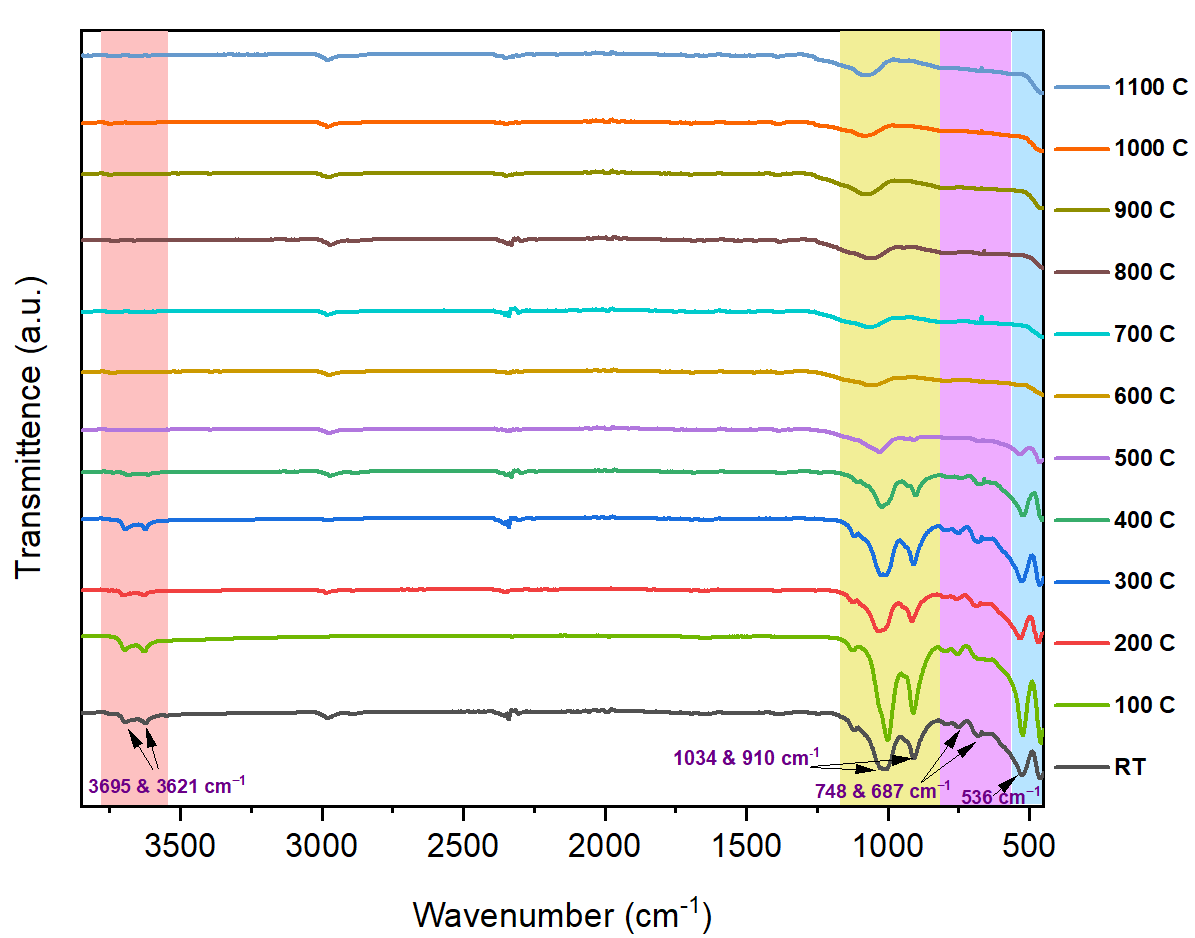


Figure S1: FT-IR spectra of the HNT samples, stretching vibration of Al-OH (red), O–Si–O and Al–O–OH (yellow), apical Si–O (purple), and Al–O–S (blue).


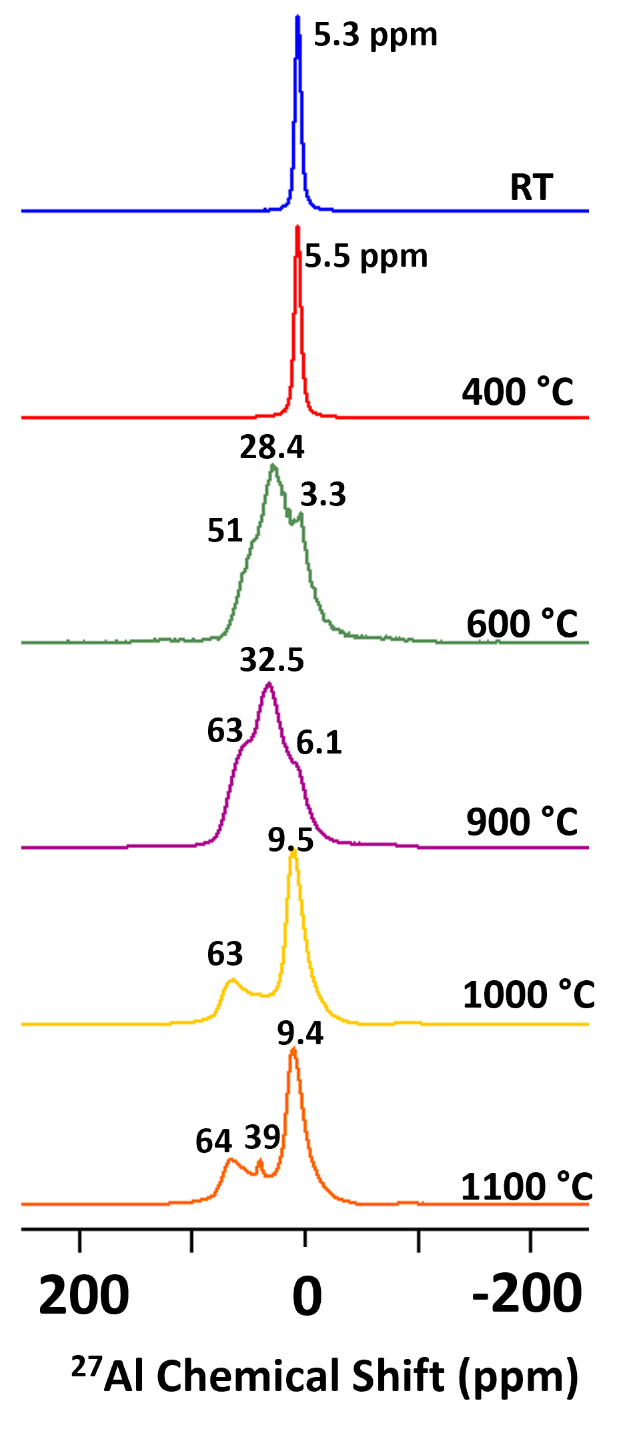


Figure S2: Solid-state 27Al NMR spectra at 12.5 kHz MAS rate of the calcined HNT samples at various temperatures.


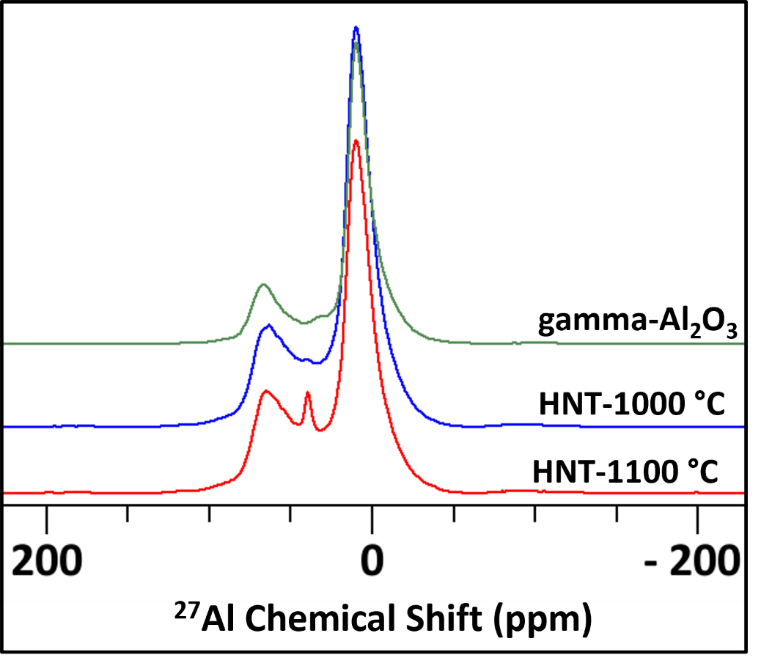


Figure S3: *Solid-state ^27^Al NMR spectra at 12.5 kHz MAS rate of γ-Al_2_O_3_ and HNT calcinated at 1000 °C and 1100 °C*


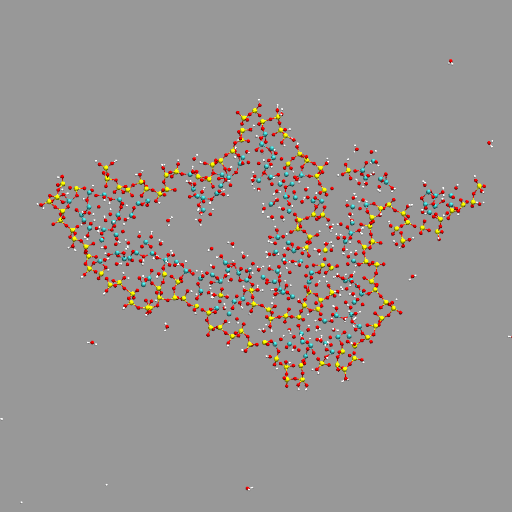


Figure S4: *HNT structure taken at 11 ps of an MD at 1100 ℃. Atom color code: Al (cyan), Si (yellow), O(red), and H (white).*
